# ﻿Low-cost, high-volume imaging for entomological digitization

**DOI:** 10.3897/zookeys.1206.123670

**Published:** 2024-07-11

**Authors:** Dirk Steinke, Jaclyn T. A. McKeown, Allison Zyba, Joschka McLeod, Corey Feng, Paul D. N. Hebert

**Affiliations:** 1 Centre for Biodiversity Genomics, University of Guelph, 50 Stone Road East, Guelph, Ontario, N1G 2W1, Canada University of Guelph Guelph Canada; 2 Department of Integrative Biology, University of Guelph, 50 Stone Road East, Guelph, Ontario, N1G 2W1, Canada University of Guelph Guelph Canada

**Keywords:** AI, Arthropoda, collections, databases, insects, machine learning, photography

## Abstract

Large-scale digitization of natural history collections requires automation of image acquisition and processing. Reflecting this fact, various approaches, some highly sophisticated, have been developed to support imaging of museum specimens. However, most of these systems are complex and expensive, restricting their deployment. Here we describe a simple, inexpensive technique for imaging arthropods larger than 5 mm. By mounting a digital SLR camera on a CNC (computer numerical control) motor-drive rig, we created a system that captures high-resolution z-axis stacked images (6960 × 4640 pixels) of 95 specimens in 30 minutes. This system can be assembled inexpensively ($1000 USD without a camera) and it is easy to set-up and maintain. By coupling low cost with high production capacity, it represents a solution for digitizing any natural history collection.

## ﻿Introduction

Advances in computational and imaging technologies have stimulated the digitization of specimens in natural history collections (Berents et al. 2010; Vollmar et al. 2010; Moore 2011; Beaman and Cellinese 2012; Mantle et al. 2012; Mathys et al. 2013; Holovachov et al. 2014; Hudson et al. 2015; Mertens et al. 2017; Hedrick et al. 2020). Because the largest collections contain millions of specimens, comprehensive digitization can be a challenge (Blagoderov et al. 2012). This is particularly true for insects, as they dominate most zoological collections (Tegelberg et al. 2014). Consequently, many digitization projects have only captured high-resolution images of a few representative specimens of each species (deWaard et al. 2019).

Projects that seek to digitize entire insect collections require automated image acquisition and processing. Because of the effort in handling individual specimens and the risk of damaging them, some digitization programs have imaged drawers of specimens (Blagoderov et al. 2012; Mantle et al. 2012; Holovachov et al. 2014). This approach has three limitations. First, resolution is often insufficient to allow examination of some morphological traits. Second, dorsal images are captured, so characters only visible with a lateral or ventral view are inaccessible. Third, this approach brings informatics challenges as the drawer image must be decomposed into its component specimen images (Blagoderov et al. 2012; Holovachov et al. 2014). In practice, most of the time required in these digitization projects is spent on image selection and metadata capture (Blagoderov et al. 2012).

Recently, several approaches have been developed to digitize individual specimens in museum collections (Heerlien et al. 2015; Tegelberg et al. 2017; Ströbel et al. 2018) or as part of community sampling and sorting procedures (Ärje et al. 2020; Wührl et al. 2021). Some of these systems generate several images per specimen to facilitate 3-D modelling (Ströbel et al. 2018) or include robotic handling of specimens to accelerate processing (Ärje et al. 2020; Wührl et al. 2021). At this time, most of these systems are elaborate and expensive.

Optimal high-throughput specimen digitization requires combining technologies in novel workflows and is largely driven by purpose (collection digitization versus one component of a multifaceted workflow). This study introduces an imaging system developed to support the specimen-centric workflow employed by the Centre for Biodiversity Genomics (CBG), Guelph, Canada to gather DNA barcode records. Because images are essential to validate DNA barcodes, the CBG photographs every specimen. Small specimens (<5 mm) are each placed into a well in a 96-well plate and are imaged with a high-resolution automated microscope system (Steinke et al. in prep.) before entering molecular analysis. Larger individuals are pinned, arrayed in Schmidt boxes, and then imaged using the digital SLR camera rig described here. This system is easy to install and was designed to provide high production capacity at low cost for operations ranging from small entomology laboratories to large natural history collections.

## ﻿Material and methods

### ﻿System hardware

**Figure 1.**
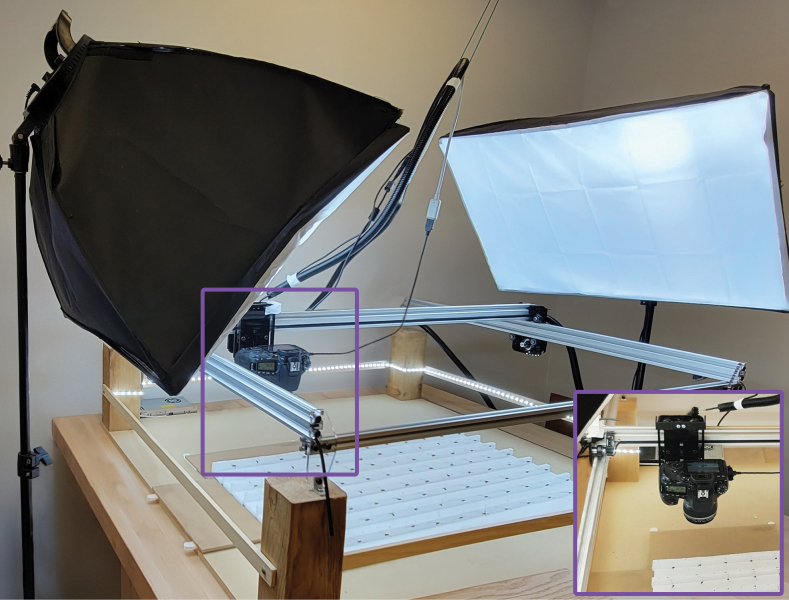
The SLR rig is placed on a heavy-base table to minimize vibration. The inset shows the actual rig area with specimens on the styrofoam base.

The SLR rig (Fig. 1) employs a Canon 90D camera (32.5 megapixel) with an EF-S 60 mm f/2.8 Macro USM lens (Canon Corp, Irvine, CA, USA). The camera is attached to an OpenBuilds Acro 1010 40” x 40” motor-drive rig (OpenBuilds, New York, NY, USA). Most components for the OpenBuilds Acro 1010 were purchased, but some components were printed (i-Fast; QIDI Technology Official, China) using 3D-models available on the OpenBuilds website (https://openbuildspartstore.com). The OpenBuilds Acro 1010 frame is screwed onto a wooden base which is placed on a sturdy table (79 cm from floor). Two 60×60 cm softbox lights (Neewer 24×24, Shenzhen) are stationed to the left and right of the SLR camera motor-drive rig at a height of 165 cm on their stands. A LED light strip is positioned along the circumference of the Acro system facing inwards (Daylight White LED Strip Light; Shenzhen Intellirocks Tech Co. Ltd., Shenzhen, China). The SLR camera is mounted 16 cm above the table’s surface and a Kimaru ACK-E6 DR-E6 DC Coupler LP-E6N Dummy Battery AC Power Adapter Kit is used to provide constant power. A USB-A to micro-USB cable connects the camera to the computer (iMac 27-inch, 8GB Ram. 3.4GHz Quad-core Intel-Core i5).

### ﻿Software control

The SLR rig is controlled by a program that employs both a python script (Suppl. material 1) and a G-code script (Suppl. material 2) through an Apple iMac operating system. The system is manually calibrated using OpenBuilds Control (OpenBuilds) integrated into the python script. The camera is controlled using the command line tool Gphoto2 (gphoto2.org). It is set to an ISO value of 100, an aperture value of f/8, and a shutter speed of 1/8s. Focus bracketing to allow z-axis stacking is set to take nine images at different levels of focus. The lens is set to auto-focus, so the first image captures the uppermost of a specimen before eight more images are automatically captured at lower focal planes to allow z-axis stacking. The nine images are combined to generate a single composite in-focus image using Helicon Focus 8 (Helicon Soft Ltd, Kharkov, Ukraine).

### ﻿Operation

**Figure 2.**
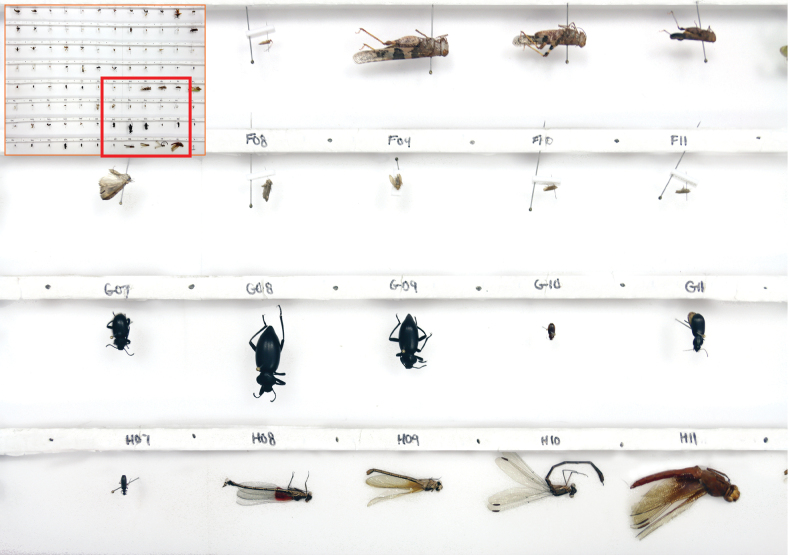
75 cm by 47 cm foam platform with pinned insects in dorsal and lateral positions.

Pinned arthropods are loaded into the SLR rig in batches of 95 after being transferred to a 75 cm by 47 cm foam platform (Fig. 2). This platform has 95 positions for specimen loading, each a slot with a depth of 1.8 cm, a length of 6 cm, and a width of 5 cm. This count ensures that all specimens in each 96-well micro-plate (95 specimens, 1 negative control) are processed as a batch. The platform is split into 8 rows, each with 12 slots (Fig. 2). Each pinned specimen is placed centrally in a slot where it can be positioned for dorsal or lateral imaging (Fig. 2). Each row has a foam strip (height = 2 cm) that facilitates lateral imaging. Specimens stored in envelopes are removed from them and placed centrally at the base of a slot.

### ﻿Data handling

Gphoto2 is used to transfer images to the computer for further processing. Z-stacked images are cropped to standardised dimensions with a 4×3 aspect ratio using a machine-learning-based cropping tool (Gharaee et al. 2023). A scale bar is added to each image using the batch action tool of Photoshop 2023 (Adobe Inc., San Jose, USA). Once edited, images are uploaded to the Barcode of Life Database System (BOLD) (Ratnasingham and Hebert 2007) where they are automatically associated with individual specimen records and the DNA barcode sequence of the photographed specimen. A python script (Suppl. material 3) generates a metadata file and compresses it together with packets of 95 images into a zip folder to meet BOLD’s requirements for image upload. Similar scripts could be developed to transfer images to any other database.

## ﻿Results and discussion

### ﻿Performance and costs

**Figure 3.**
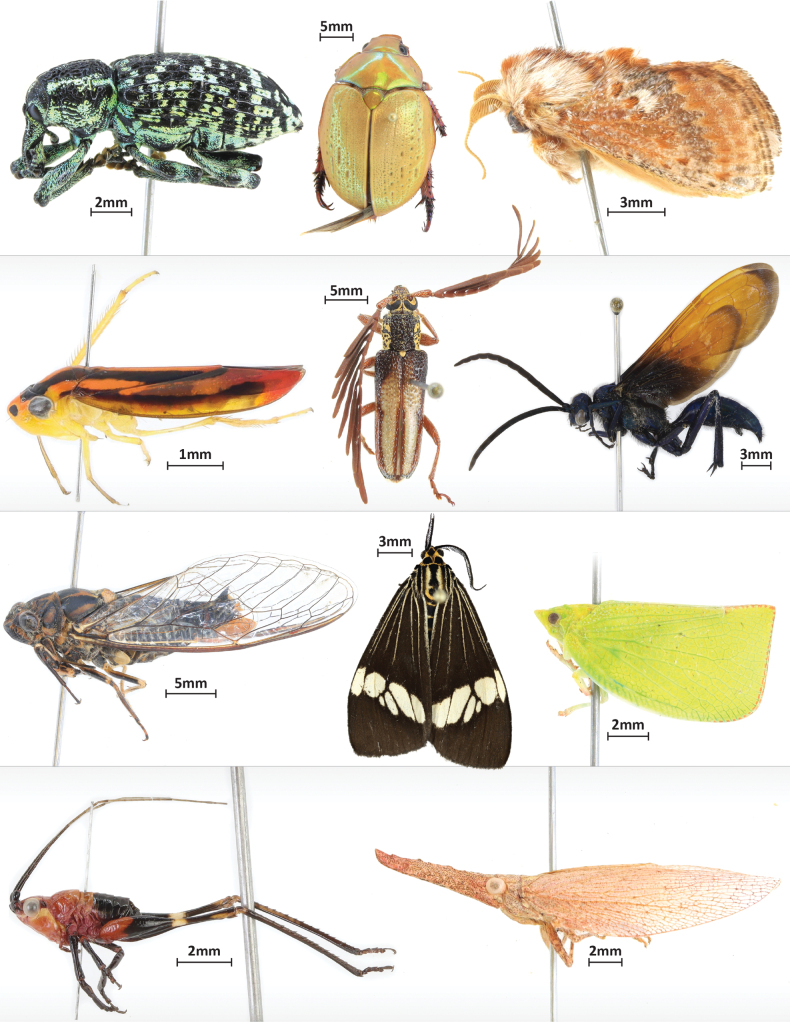
Panel of example images taken with the SLR rig.

Fig. 3 shows a selection of specimens and their sizes. When using a Canon 90D with the described settings, the resulting image is 6960 × 4640 pixels before cropping. This translates into an average size of 9.5MB for a jpg-file.

The CNC motor activates at a pre-set time, which depends upon the distance between each slot and the dwell time (3.75 s) at each stop, allowing the camera to take nine images and transfer them to the computer. Operating in this mode, the SLR rig images 95 specimens in 30 min and the stacking software requires another 11.5 min to process these images, but this usually occurs while the next batch is being photographed. The transfer of pinned specimens to and off the foam platform takes about 15 min each and is done while the SLR rig is running another batch. The time required to crop and edit each batch varies (15–30 min) with the type of specimens. Operated by one staff member, the SLR rig can image 4000 specimens in a week. The CBG’s system has now imaged more than 250,000 specimens and the sole maintenance involved the replacement of a wire leading to one motor.

The SLR rig cost of $4500 USD reflects three main components: 1) CNC machine kit ($1000), 2) Apple computer ($1000), and 3) Canon 90D including lens ($2500). Costs can be reduced by replacing the computer with a raspberry pi ($100), but under heavy usage (40 h a week), it will need replacement every six months. Less expensive cameras can be used if they can be controlled with gphoto2. They do have a lower resolution (12–20 megapixels) than the Canon 90D (32.5 megapixels), but this resolution is adequate for many applications. However, it is important to select a camera with a depth stacking function such as focus bracketing (e.g., Canon PowerShot G7 X Mark II, $900; Olympus OM-D E-M1 Mark II & III, $920 for body). By careful selection of components, the overall cost can be reduced to about $2000 (using the CNC kit, a raspberry pi, and a low budget point and shoot camera with depth stacking). The light setup can also vary in cost. The Neewer 24×24 Softbox pair used in our study costs about $150 but it can be replaced by LED strips (~$20) attached to the inner part of the CNC frame. Plastic components for the OpenBuilds Acro 1010 are freely available as 3D models so users can modify and 3D-print custom components if such a system is available. One modification made to our SLR rig was the addition of bumpers and a triangular structure to improve wire management during operation (Suppl. materials 4, 5).

### ﻿Adjustments

The SLR rig can image a wide variety of specimens by adjusting settings as described in this section. The distance between the camera and the specimen dictates the size of the image (focal distance from base = 16 cm). The frame size varies by 0.5 cm in both directions depending upon the depth of the focus point determined by the auto-focus program. This limits the size range of specimens which can be imaged (5–45 mm). As each slot on the platform is designed to fit the camera frame, no specimen should overlap an adjacent space because the cropping tool is likely to malfunction. However, larger specimens can be imaged if the distance between the camera and the specimen is increased as this enlarges the size of the frame. Conversely, reducing the camera-specimen distance decreases the size of the frame, allowing smaller specimens (down to 2 mm using the 60 mm macro lens in our setup) to be imaged. Any change in the camera’s operating height is difficult with the described setup as it requires remounting the camera at a higher or lower position on its mount or the exchange of the legs mounted to the rig frame. Future optimization could incorporate legs capable of height adjustment.

Background colour and light settings can also be modified to improve image quality. Dark backgrounds improve the contrast for dark specimens, helping to highlight otherwise subtle features and also help to contrast pale specimens that blend into a white background. To make this adjustment, a second platform can be made of dark foam, or dark strips can be temporarily added to the existing platform. As lighting and whitening settings on the camera must be adjusted to accommodate the change in background colour, all 95 slots must have the same background.

The number of images taken of each specimen can be adjusted with the depth stack function on the camera. Increasing the image count expands the depth range in focus, but increases the time required to capture photographs and to process them in Helicon Focus. The dwell time of the CNC motor system would need to be extended to allow more images to be taken before the camera moves. Conversely, imaging and processing times can be reduced by reducing the number of images taken per specimen. Experimentation with sets of specimens in the target group is the best way to optimize the number of images taken.

Although this CBG’s SLR system is primarily used with pinned insects, it is effective in imaging other specimens (e.g., soft-bodied invertebrates in liquid preservatives). In the latter case, the foam platform is simply replaced by a grid structure that holds each specimen vial (Mendez et al. 2018). Focal distance and stack depth often need to be adjusted in such cases (Mendez et al. 2018).

### ﻿Limitations

Generating an image with enough resolution to allow species identification can be difficult with any automated system given the manifold differences in shape and size of specimens (Blagoderov et al. 2012). Very large individuals that exceed the standard stacking depth can cause the auto-focus program to return an out-of-focus image. The auto-focus function is also vulnerable to vertical protrusions, especially if they contrast with the background. In such cases, the depth stack may begin above the organism’s body plane leading to a blurred image. For winged insects, such as Lepidoptera, variation in wing orientation can lead the wingspan exceeding the range of the image stack. In such cases, the resulting image may show a focused wing with an out-of-focus body or an in-focus body with a blurred wing. In such cases, a slight change in the angle at which a specimen is positioned can greatly improve image quality but a switch from lateral to dorsal view is sometimes required. Because reorienting a few specimens requires recapturing an entire set of images, it is often more time-effective to simply accept few imperfect images (Chapman 2005; Ahl et al. 2023). Such specimens could be imaged separately using any setup.

At the CBG, specimens are usually imaged before they are labelled. When labelled specimens are imaged, a small white piece of paper with a slit in the middle is used to cover labels, allowing images of small specimens to remain sharp when cropped. Alternatively, the labels can be removed and reattached to the specimen after photography.

## ﻿Conclusion

The present SLR rig was designed to photograph terrestrial arthropods that were being analyzed to construct DNA barcode reference libraries. About 90% of these specimens are small enough to be imaged within 96-well plates, but the remainder must be pinned. As the CBG currently barcodes three million specimens annually, it was essential to develop a system capable of imaging the larger specimens in a cost-effective way. This led to the present solution, which can be acquired for $2000–4500 USD depending on the choice of camera and controller and generates almost 200 high-resolution specimen images per hour.

As the CBG’s SLR rig has performed reliably for 2.5 years of heavy use (12h/day), this system is ideal for deployment in settings remote from technical support. Because of its capacity to rapidly generate large numbers of high-quality digital images for online databases, it is also an asset for any large specimen collection.

## ﻿Additional information

### Author ORCIDs

Dirk Steinke https://orcid.org/0000-0002-8992-575X

Jaclyn T. A. McKeown https://orcid.org/0009-0005-7193-2643

Joschka McLeod https://orcid.org/0000-0002-7503-1835

Corey Feng https://orcid.org/0009-0007-7630-7884

Paul D. N. Hebert https://orcid.org/0000-0002-3081-6700

### Data availability

All of the data that support the findings of this study are available in the main text or Supplementary Information.

## Supplementary materials

Supplementary material 1 SLR Rig controlling script Data type py https://binary.pensoft.net/file/1089601This dataset is made available under the Open Database License (http://opendatacommons.org/licenses/odbl/1.0/). The Open Database License (ODbL) is a license agreement intended to allow users to freely share, modify, and use this Dataset while maintaining this same freedom for others, provided that the original source and author(s) are credited.Dirk Steinke, Jaclyn T. A. McKeown, Allison Zyba, Joschka McLeod, Corey Feng, Paul D. N. Hebert

Supplementary material 2 G-code control script Data type gcode https://binary.pensoft.net/file/1089602This dataset is made available under the Open Database License (http://opendatacommons.org/licenses/odbl/1.0/). The Open Database License (ODbL) is a license agreement intended to allow users to freely share, modify, and use this Dataset while maintaining this same freedom for others, provided that the original source and author(s) are credited.Dirk Steinke, Jaclyn T. A. McKeown, Allison Zyba, Joschka McLeod, Corey Feng, Paul D. N. Hebert

Supplementary material 3 Image packaging and upload to BOLD Data type py https://binary.pensoft.net/file/1089603This dataset is made available under the Open Database License (http://opendatacommons.org/licenses/odbl/1.0/). The Open Database License (ODbL) is a license agreement intended to allow users to freely share, modify, and use this Dataset while maintaining this same freedom for others, provided that the original source and author(s) are credited.Dirk Steinke, Jaclyn T. A. McKeown, Allison Zyba, Joschka McLeod, Corey Feng, Paul D. N. Hebert

Supplementary material 4 File for 3D printing of bumpers used in system Data type x3d https://binary.pensoft.net/file/1089604This dataset is made available under the Open Database License (http://opendatacommons.org/licenses/odbl/1.0/). The Open Database License (ODbL) is a license agreement intended to allow users to freely share, modify, and use this Dataset while maintaining this same freedom for others, provided that the original source and author(s) are credited.Dirk Steinke, Jaclyn T. A. McKeown, Allison Zyba, Joschka McLeod, Corey Feng, Paul D. N. Hebert

Supplementary material 5 File for 3D printing of triagular wire management piece Data type x3d https://binary.pensoft.net/file/1089605This dataset is made available under the Open Database License (http://opendatacommons.org/licenses/odbl/1.0/). The Open Database License (ODbL) is a license agreement intended to allow users to freely share, modify, and use this Dataset while maintaining this same freedom for others, provided that the original source and author(s) are credited.Dirk Steinke, Jaclyn T. A. McKeown, Allison Zyba, Joschka McLeod, Corey Feng, Paul D. N. Hebert

## References

[B1] Ärje J Melvad C Rosenhøj Jeppesen M Agerskov Madsen S Raitoharju J Strandgård Rasmussen M Iosifidis A Tirronen V Gabbouj M Meissner K Høye TT (2020) Automatic image-based identification and biomass estimation of invertebrates. Methods in Ecology and Evolution 11(8): 922–931. 10.1111/2041-210X.13428

[B2] Beaman RS Cellinese N (2012) Mass digitization of scientific collections: New opportunities to transform the use of biological specimens and underwrite biodiversity science. ZooKeys 209: 7–17. 10.3897/zookeys.209.3313 PMC3406463 22859875

[B3] Berents P Hamer M Chavan V (2010) Towards demand-driven publishing: Approaches to the prioritization of digitization of natural history collection data. Biodiversity Informatics 7: 113–119. 10.17161/bi.v7i2.3990

[B4] Blagoderov V Kitching IJ Livermore L Simonsen TJ Smith VS (2012) No specimen left behind: Industrial scale digitization of natural history collections. ZooKeys 209: 133–146. 10.3897/zookeys.209.3178 PMC3406472 22859884

[B5] Chapman A (2005) Principles of Data Quality. Global Biodiversity Information Facility. 10.15468/doc.jrgg-a190

[B6] deWaard JR Ratnasingham S Zakharov EV Borisenko AV Steinke D Telfer AC Perez KHJ Sones JE Young MR Levesque-Beaudin V Sobel CN Abrahamyan A Bessonov K Blagoev G deWaard SL Ho C Ivanova NV Layton KKS Lu L Manjunath R McKeown JTA Milton MA Miskie R Monkhouse N Naik S Nikolova N Pentinsaari M Prosser SWJ Radulovici AE Steinke C Warne CP Hebert PDN (2019) A reference library for Canadian invertebrates with 1.5 million barcodes, voucher specimens, and DNA samples. Scientific Data 6(1): 308. 10.1038/s41597-019-0320-2 31811161 PMC6897906

[B7] Gharaee Z Gong Z Pellegrino N Zarubiieva I Haurum JB Lowe SC McKeown JTA Ho CCY McLeod J Wei YC Agda J Ratnasingham S Steinke D Chang AX Taylor GW Fieguth P (2023) A step towards worldwide biodiversity assessment: The BIOSCAN-1M insect dataset. Advances in Neural Information Processing Systems 37.

[B8] Hedrick BP Heberling JM Meineke EK Turner KG Grassa CJ Park DS Kennedy J Clarke JA Cook JA Blackburn DC Edwards SV Davis CC (2020) Digitization and the future of natural history collections. Bioscience 70(3): 243–251. 10.1093/biosci/biz163

[B9] Heerlien M van Leusen J Schnörr S de Jong-Kole S Raes R van Hulsen K (2015) The natural history production line: An industrial approach to the digitization of scientific collections. ACM Journal on Computing and Cultural Heritage 8(1): 1–11. 10.1145/2644822

[B10] Holovachov O Zatushevsky A Shydlovsky I (2014) Whole-drawer imaging of entomological collections: Benefits, limitations and alternative applications. Journal of Conservation & Museum Studies 12(1): 9. 10.5334/jcms.1021218

[B11] Hudson LN Blagoderov V Heaton A Holtzhausen P Livermore L Price BW van der Walt S Smith VS (2015) Inselect: Automating the digitization of natural history collections. PLoS one 10: e0143402. 10.1371/journal.pone.0143402 PMC4658125 26599208

[B12] Ahl LI Bellucci L Brewer P Gagnier P-Y Hardy HM Haston EM Livermore L De Smedt S Enghoff H (2023) Digitisation of natural history collections: criteria for prioritization. Research Ideas and Outcomes 9: e114548. 10.3897/rio.9.e114548

[B13] Mantle BL Salle JL Fisher N (2012) Whole-drawer imaging for digital management and curation of a large entomological collection. ZooKeys 209: 147–163. 10.3897/zookeys.209.3169 PMC3406473 22859885

[B14] Mathys A Brecko J Semal P (2013) Comparing 3D digitizing technologies: What are the differences? Digital Heritage International Congress. Marseille, 201–204. 10.1109/DigitalHeritage.2013.6743733

[B15] Mendez PK Lee S Venter CE (2018) Imaging natural history museum collections from the bottom up: 3D print technology facilitates imaging of fluid-stored arthropods with flatbed scanners. ZooKeys 795: 49–65. 10.3897/zookeys.795.28416 PMC6232236 30429657

[B16] Mertens JEJ Roie MV Merckx J Dekoninck W (2017) The use of low-cost compact cameras with focus stocking functionality in entomological digitization projects. ZooKeys 712: 141–154. 10.3897/zookeys.712.205055 PMC5674212 29134038

[B17] Moore W (2011) Biology needs cyber-infrastructure to facilitate specimen-level data acquisition for insect and other hyperdiverse groups. ZooKeys 147: 479–486. 10.3897/zookeys.147.1944 PMC3286256 22371672

[B18] Ratnasingham S Hebert PDN (2007) BOLD: the barcode of life data system (http://www.barcodinglife.org). Molecular Ecology Notes 7(3): 355–364. 10.1111/j.1471-8286.2007.01678.x PMC1890991 18784790

[B19] Ströbel B Schmelzle S Blüthgen N Heethoff M (2018) An automated device for the digitization and 3D modelling of insects, combining extended-depth-of-field and all-side multi-view imaging. ZooKeys 759: 1–27. 10.3897/zookeys.759.24584 PMC5968080 29853774

[B20] Tegelberg R Mononen T Saarenmaa H (2014) High-performance digitization of natural history collections: Automated imaging lines for herbarium and insect specimens. Taxon 63(6): 1307–1313. 10.12705/636.13

[B21] Tegelberg R Kahanpää J Karppinen J Mononen T Wu Z Saarenmaa H (2017) Mass digitization of individual pinned insects using conveyor-driven imaging. 2017 IEEE 13^th^ International Conference on e-Science (e-Science): 523–527. 10.1109/eScience.2017.85

[B22] Vollmar A Macklin JA Ford LS (2010) Natural history specimen digitization: Challenges and concerns. Biodiversity Informatics 7(2): 93–113. 10.17161/bi.v7i2.3992

[B23] Wührl L Pylatiuk C Giersch M Lapp F von Rintelen T Balke M Schmidt S Cerretti P Meier R (2021) DiversityScanner: Robotic discovery of small invertebrates with machine learning methods. BioRxiv preprint. 10.1101/2021.05.17.444523 34863029

